# Genome-Wide Analysis of Aquaporin Gene Family in *Triticum turgidum* and Its Expression Profile in Response to Salt Stress

**DOI:** 10.3390/genes14010202

**Published:** 2023-01-12

**Authors:** Mahnaz Yaghobi, Parviz Heidari

**Affiliations:** Faculty of Agriculture, Shahrood University of Technology, Shahrood 3619995161, Iran

**Keywords:** water channel, abiotic stresses, wheat genes, post-translation modification, gene expression

## Abstract

During the response of plants to water stresses, aquaporin (AQP) plays a prominent role in membrane water transport based on the received upstream signals. Due to the importance of the AQP gene family, studies have been conducted that investigate the function and regulatory system of these genes. However, many of their molecular aspects are still unknown. This study aims to carry out a genome-wide investigation of the AQP gene family in *Triticum turgidum* using bioinformatics tools and to investigate the expression patterns of some members in response to salt stress. Our results show that there are 80 *TtAQP* genes in *T. turgidum*, which are classified into four main groups based on phylogenetic analysis. Several duplications were observed between the members of the TtAQP gene family, and high diversity in response to post-translational modifications was observed between TtAQP family members. The expression pattern of *TtAQP* genes disclosed that these genes are primarily upregulated in response to salt stress. Additionally, the qPCR data revealed that *TtAQPs* are more induced in delayed responses to salinity stress. Overall, our findings illustrate that TtAQP members are diverse in terms of their structure, regulatory systems, and expression levels.

## 1. Introduction

Aquaporin proteins (AQPs) include a group of major intrinsic proteins (MIPs) that facilitate the transport of water and other small neutral molecules through the cell membrane by forming channels in the membrane, and play an important role in plants’ growth and responses to abiotic stress [1,2]. AQP channels facilitate bidirectional flow across a concentration gradient in all cells [3]. In addition, some AQP isoforms play the role of peroxyporins and contribute to cellular redox signaling by transporting hydrogen peroxide [4]. Moreover, AQPs in plants have multiple functions, such as the hydraulic control of plant tissue, as well as seed germination and the emergence of lateral roots [2]. Based on structural analysis, AQP family genes can be divided into five main evolutionary subfamilies in plants, including plasma membrane intrinsic proteins (PIP); tonoplast internal proteins (TIP); Nodulin-26 intrinsic proteins such as NIPs, which are small major intrinsic proteins (SIPs); and X intrinsic proteins (XIPs) [5]. Plant AQPs have two fundamental factors; they are extraordinary divers [6], and some are multifunctional channel proteins that allow some small neutral solutes to cross the cell membrane, such as glycerol, CO_2_, ammonia (NH _3_), urea, boron, and hydrogen peroxide [7].

In recent years, the function of AQPs has been investigated in relation to plant responses to biotic and abiotic stresses [8,9,10,11]. It has been shown that AQP can improve plant tolerance to abiotic stresses (such as drought, osmosis, cold, salt, and high temperature) [12]. Furthermore, previous studies show that AQP plays a positive role in responses to biotic stresses. It has also been found that the expression of *AQP* genes differs depending on the organs and tissues, as well as plant growth regulators and abiotic stress such as drought, heat, and cold [13,14]. Between AQP subfamilies, the function of PIPs and TIPs, which have high transporter activity, is investigated more under adverse conditions [15]. The downregulation of PIPs and TIPs was reported in response to drought and salt stress [1,16,17,18]. Additionally, it was stated that the NIP subfamily is the only AQP able to transport Si (silicon) that can improve plant tolerance in response to various environmental stresses [5,19].

Today, cereals are considered major crops, and wheat is considered a strategic crop among cereals across the globe. Wheat genotypes exist in three ploidy levels, which include diploid, tetraploid, and hexaploid [20]. Durum wheat, *T. turgidum* L. ssp. *durum* (Desf.), is a tetraploid wheat (2n = 4x = 28). *T. turgidum* is best adapted to areas with a relatively dry climate with warm days and cool nights during the growing season (corresponding to a Mediterranean and temperate climate) [21]. As mentioned above, AQPs are critical in transporting water and maintaining cell balance in response to stresses such as drought and salinity. However, the structure and function of AQP gene family members in response to salinity are largely unknown in *T. turgidum*. In the current study, we aimed to identify and characterize AQPs in *T. turgidum* (TtAQPs), and the expression levels of several *TtAQPs* were evaluated at different salinity concentrations. As the first report on this topic, new perspectives related to the structure, process of evolution, and function of members of this gene family are presented.

## 2. Materials and Methods

### 2.1. Identification and Sequence Analysis of TtAQPs

To identify the *AQP* genes in *T. turgidum* L. ssp. *durum* (Desf.) (*TtAQPs*) [22], the protein sequences of putative AQPs in *Arabidopsis* (AtAQPs) and rice (OsAQPs) were applied as the query sequences against the complete protein sequence of *T. turgidum* using the BLASTP tool of the EnsemblPlants database (Accessed on 24 November 2022) [23]. In the next step, the extracted sequences were confirmed using the Conserved Domains Database (CDD) (Accessed on 24 November 2022) [24], and Pfam [25]. Then, short sequences (less than 150 amino acids in length) were removed from the list. The identified sequences were analyzed using the Expasy website and their physicochemical properties, such as molecular weight (MW), isoelectric point (pI), instability index, and GRAVY index were estimated using the ProtParam tool [26].

### 2.2. Investigating Phylogenetics of TtAQPs

To study the relationships of TtAQP proteins in *T. turgidum*, a phylogenetic tree was constructed with the complete protein sequences of the TtAQP gene family members along with their orthologues from *Arabidopsis*, rice, barley, corn, and sorghum plants. *AQP* genes were identified in Arabidopsis (*Arabidopsis thaliana*), barley (*Hordeum vulgare*), corn (*Zea mays*), and rice (*Oryza sativa* japonica) using the EnsemblPlants database, as was *T. turgidum*. The sequence of AQP proteins was aligned using the ClustalW program using an online tool, Clustal Omega [27]. Then, the phylogenetic tree of AQPs was constructed based on the maximum likelihood (ML) method with 1000 bootstrap replications using the IQ tree website [28]. The iTOL tool [29] was used to illustrate the phylogenetic tree. Additionally, ten conserved motifs in TtAQPs were identified using the MEME motif finder tool [30] based on the default setting.

### 2.3. Determining the Duplication Genes and Estimating Ka and Ks Values

The duplication events between *TtAQP* genes were identifiedbased on similarity, more than 80% between pairs of *TtAQP* genes [31]. In addition, synonymous (Ks) and non-synonymous (Ka) values at each site among pairs of duplicated genes were calculated using TBtools software (v1.068) [32]. Then, the Ka/Ks ratio was estimated to identify the effect of evolution pressure on the function of duplicated genes. The division time of pairs of duplicated *TtAQP* genes was estimated using the synonymous mutation rate of substitution λ per synonymous site per year, as T = (Ks/2λ (λ = 6.5 × 10^−9^)) × 10^−6^ [33].

### 2.4. Prediction of Phosphorylation Site in TtAQP Proteins

Phosphorylation is one of the most important types of post-translational modification. The sites of phosphorylation are serine, tyrosine, and threonine residues on proteins. The potential phosphorylation regions of TtAQP proteins were predicted through the NetPhos 3-1 site, with a potential value higher than 0.80 [34].

### 2.5. Prediction of 3D Structures and Pocket Sites of AQP Proteins

The three-dimensional structure of TtAQP proteins was predicted using the Phyre2 database [35]. The validity of the predicted protein model was evaluated through Ramachandran plot analysis [36]. The analysis of ligand binding regions (pocket sites) in the predicted protein models was also performed through the Phyre investigator tool of the Phyre2 server.

### 2.6. TtAQP Gene Promoter Analysis

To identify the regulatory regions in the promoter region, a region 1500 nucleotides upstream of the *TtAQP* genes was investigated as the promoter region. The sequence of the promoter region was analyzed to recognize the putative cis-regulatory elements using the PlantCARE database [37]. Finally, the identified cis-regulatory elements were grouped based on their functions.

### 2.7. Interaction Network of TtAQPs

In the present study, we constructed an interaction network of TtAQP proteins based on their homologs in *T. aestivum* using the String database (https://string-db.org, accessed on 24 November 2022). Additionally, gene ontology (GO) enrichment analysis of proteins presented in the network was performed. To identify the significant GO terms, the FDR (false discovery rate) was adjusted to <0.01.

### 2.8. Plant Materials and Treatments

Seeds of *T. turgidum* L. ssp. *durum* (Desf.) cultivar Yavaros were sterilized with 3% sodium hypochlorite for 10 min. Then, they were cultivated in pots containing sterilized perlite and peat moss (2:1). Five seeds were planted in each pot, and after germination, three of the best seedlings were left in each pot. The cultivation conditions were kept with a photoperiod of 16 h of light and 8 h of darkness and a temperature of 25 °C. Forty-day-old seedlings were subjected to salt treatment via irrigation. In the current study, two salt concentrations, 150 and 250 mM of NaCl, were applied twice at 24 h intervals. Some pots were also considered as controls (without using salt stress). After applying salt stress, the leaves of seedlings were sampled at different exposure periods of 6, 24, and 72 h. The collected samples were immediately placed in liquid nitrogen, and then, were transferred to a −80 °C freezer for other analyses.

### 2.9. Primer Design to Study Gene Expression

To investigate gene expression, six *TtAQP* genes, including *TtAQP18*, *TtAQP29*, *TtAQP34*, *TtAQP79TtAQP58*, and *TtAQP42*, were selected based on the phylogenetic result. The primers of the selected *TtAQP* genes were designed based on the coding region (CDS) using Primer3 online software [38]. In this study, *Actin7* was used as a reference gene (Table S1).

### 2.10. RNA Extraction and Real-Time PCR

An RNX plus kit (Sinaclon, Tehran, Iran) was used for RNA extraction based on the manufacturer’s instructions. The quality and quantity of the extracted RNA were checked using a NanoPhotometer (Implen N50, IMPLEN, München, Germany). Complementary DNA (cDNA) was synthesized by reverse transcriptase (Roche, Mannheim, Germany) based on the manufacturer’s protocols. The expression levels of *TtAQP* genes were investigated using Maxima SYBR Green/ROX qPCR (quantitative real-time PCR) Master Mix (Thermo Fisher, Illkirch-Graffenstaden, France) based on the manufacturer’s protocol and ABI Step One. The cycling patterns of qPCR were 95 °C for 10 min, then 35 cycles at 95 °C for 15 s, and 60 °C for 60 s. The relative expression of each gene was calculated using the delta delta Ct method [39]. The expression difference between the treatments and the control sample was calculated based on a t-test. All experiments were performed in three biological replicates.

## 3. Results

### 3.1. Identification and Characterization of AQPs

In the current study, 80 putative *TtAQP* genes were identified in the genome of *T. turgidum*. Detailed information on the 80 *TtAQP*s is provided in Table S2. In addition, TtAQPs were compared with their orthologues in *A. thaliana*, *H. vulgare*, *O. sativa*, and *Z. mays* based on their physicochemical properties (Table 1). It was observed that the AQPs of the studied plants varied based on their physicochemical properties. In *T. turgidum*, TtAQPs varied between 157 (TtAQP61) and 392 (TtAQP27) amino acids, and their pI ranged between 4.65 (TtAQP42) and 11.05 (TtAQP09). The exon number of the deduced proteins varied from 1 (most members of the PIP2 subfamily) to 6 and their MWs ranged from 16.7 (TtAQP42) to 41.5 (TtAQP27) kDa. In addition, the GRAVY value of TtAQPs varied between −0.46 and 0.94, and 96% of the deduced proteins were predicted as stable proteins (Table 1 and Table S2).

### 3.2. Evolutionary Analysis

**Figure 1:**
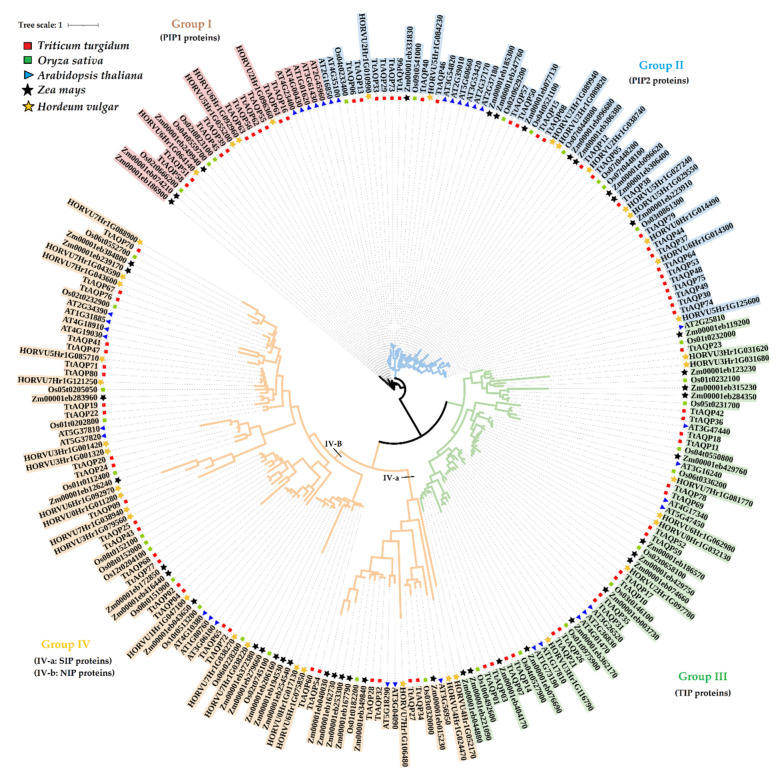
Phylogenetic analysis of AQP gene family in *T. turgidum* (TtAQPs), *O. sativa* (starting with Os), *Z. mays* (starting with Zm), *H. vulgare* (starting with HOR), and *A. thaliana* (starting with AT).

**Figure 2:**
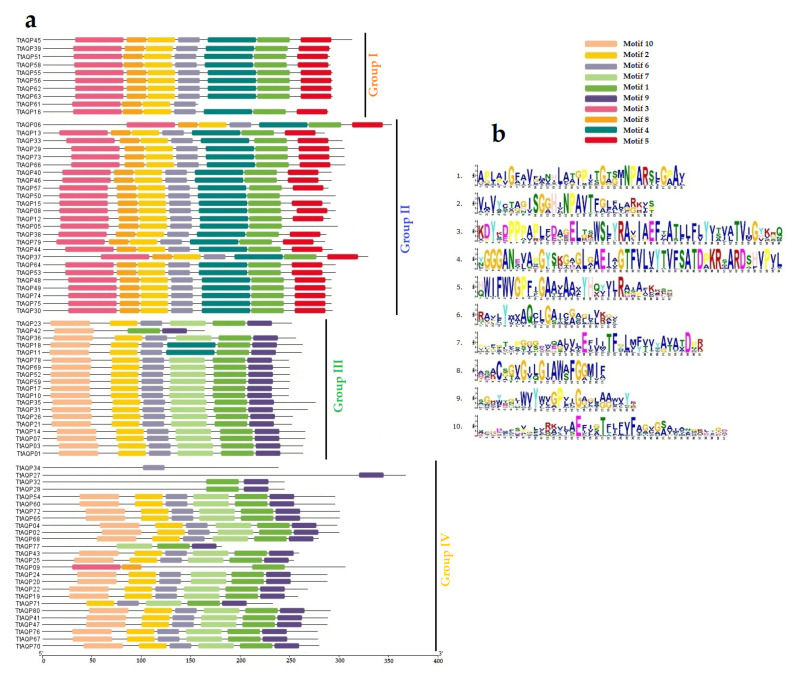
Conserved motifs in TtAQP proteins. Distribution of 10 conserved motifs in TtAQPs in four groups based on phylogenetic analysis (**a**), and conserved logos of identified motifs (**b**).

To investigate the relationships of TtAQPs, 237 AQP proteins, including 80 *T. turgidum* AQPs, 39 *H. vulgare* AQPs (HvAQPs), 35 *Arabidopsis* AQPs (AtAQPs), 39 rice AQPs (OsAQPs), and 44 maize AQPs (ZmAQPs), were used to design a phylogenetic tree. According to the phylogenetic tree, all AQPs were classified into four main groups (Figure 1). All PIP1 proteins were located in group 1, PIP2 proteins were placed in group 2, and TIPs were in group 3. In addition, SIPs were located in group 4-a, and NIPs were placed in group 4-b. Ten TtAQPs were present in group 1 with five TtAQPs, four HvAQPs, three OsAQPs, and three ZmAQPs. Additionally, 25 TtAQPs with 8 AtAQPs, 10 HvAQPs, 8 OsAQPs, and 9 ZmAQPs were located in group 2. In addition, 19 TtAQPs with 10 AtAQPs, 7 HvAQPs, 10 OsAQPs, and 14 ZmAQPs were present in group 4. Finally, 26 TtAQPs with 12 AtAQPs, 19 HvAQPs, 14 OsAQPs, and 18 ZmAQPs were located in group 4. Based on the phylogenetic analysis, TtAQPs showed a close relationship with their orthologues in barley. Moreover, AQPs from group 1 showed more genetic distance from the other members. Ten conserved domains were identified in TtAQPs (Figure 2). Motifs 3, 4, 5, and 8 were present in proteins from groups 1 and 2, including PIP1 and PIP2 proteins. Additionally, motifs 10, 7, and 9 were observed in proteins groups 3 and 4. These conserved motifs can be used to identify each subfamily of TtAQPs.

### 3.3. Duplication Events in TtAQPs

**Figure 3:**
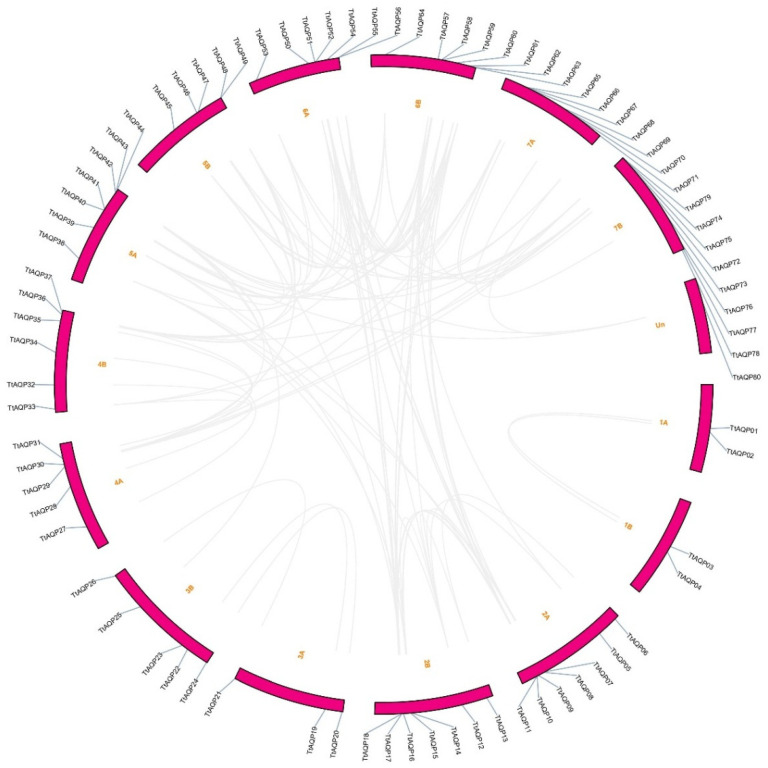
The location of *TtAQP* genes in the genome of *T. turgidum*. Duplicated genes are connected with gray lines.

**Figure 4:**
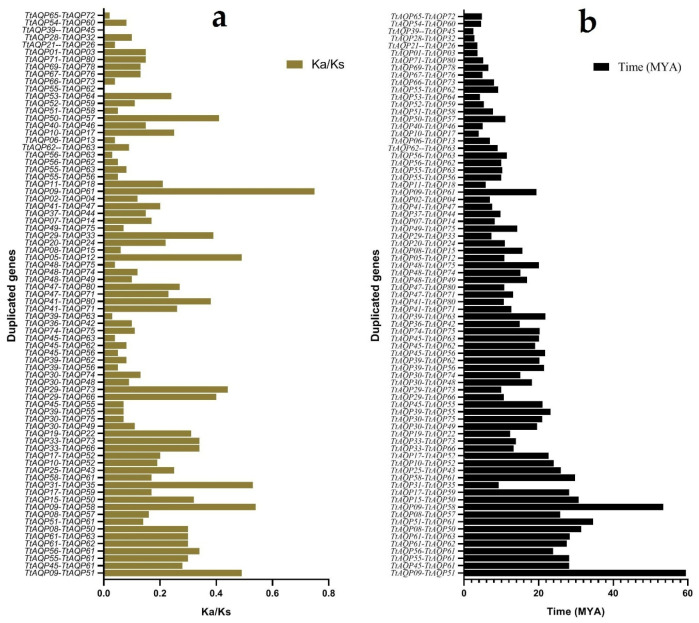
The Ka/Ks ratio for duplicated *TtAQP* genes (**a**), and time of divergence (MYA: million years ago) of the duplicated *TtAQP* genes (**b**).

The genomic distribution of each *TtAQP* was investigated in the *T. turgidum* genome (Figure 3). Eighty *TtAQP* genes were located on 14 chromosomes and one chromosome was unknown (UN). In terms of the gene distribution on chromosomes, Chr6B and Chr7B had the highest distribution and frequency, with eight *TtAQP* genes on each. After these two chromosomes, Chr2A, Chr2B, Chr5A, Chr6A, and Chr7A were more abundant, with seven *TtAQP* genes on each. In addition, Chr1A and Chr1B, each with two genes, included the lowest number of genes. Finally, a *TtAQP* gene was located in unknown chromosomal positions. These results determined that *TtAQPs* are not uniformly distributed on *T. turgidum* chromosomes, and probably during the evolution and polyploidy processes, these genes increased randomly. Additionally, the investigation of the duplication process between *TtAQP* genes showed that many duplications occurred between members in the TtAQP family during evolution (Figure 3 and Table S3). Accordingly, 5% tandem duplication was observed for the TtAQP family (Table S3), and most duplications were of the segmental type, which indicates gene transfer and a change in the chromosome set. Additionally, based on the ka/ks ratio, the duplicated events between TtAQP family members were under purifying selection, which caused a decrease in diversity (Figure 4a). Moreover, the first duplication probably occurred around 59 million years ago between *TtAQP09* and *TtAQP51* (Figure 4b).

### 3.4. Protein Structure Analysis of TtAQPs

**Figure 5:**
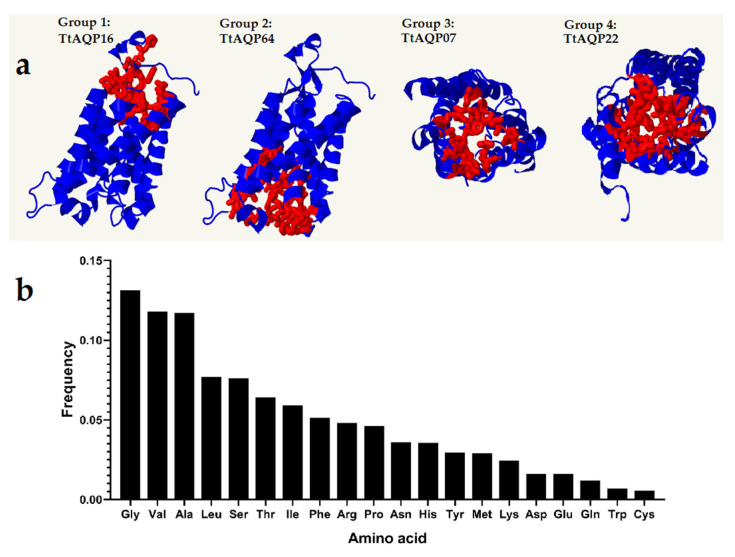
Three-dimensional structure analysis of TtAQP proteins (**a**), and frequency of amino acids present in binding sites of TtAQPs (**b**). Red balls show the binding sites in the structure of selected proteins.

The prediction results of the three-dimensional structure of TtAQP proteins showed that these proteins have different interaction areas (Figure 5a). Based on their three-dimensional structure, TtAQPs from group 1 and group 2, including PIP proteins, had similar structures. However, they differed in their location and type of binding region (pocket sites) (Figure 5a,b). The abundance of amino acids in the interaction and binding regions of TtAQP proteins was also determined (Figure 5b). The amino acids, including glycine, alanine, and valine, had the highest frequency in the binding region of TtAQP proteins. These areas could be considered more in functional studies.

### 3.5. Phosphorylation Analysis of TtAQPs

**Figure 6:**
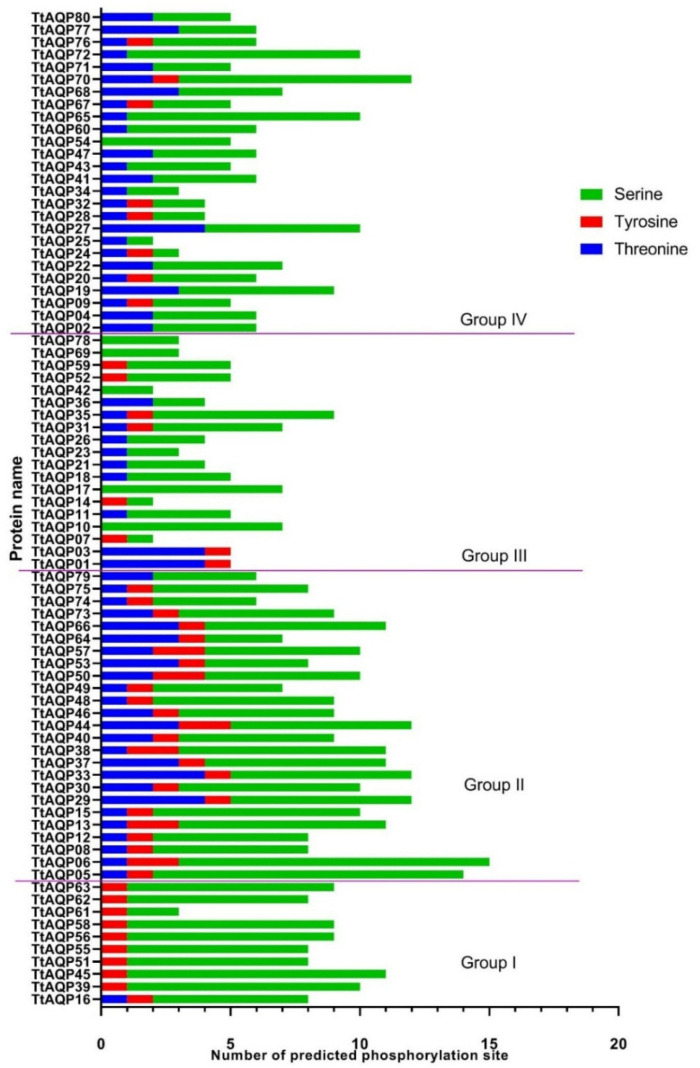
Prediction of phosphorylation site in TtAQPs based on three amino acids, including serine, tyrosine, and threonine. The potential value is >0.80.

The phosphorylation regions, in terms of the three amino acids serine, tyrosine, and threonine in TtAQP proteins, were predicted. According to the analysis, it was found that the amount of serine compared to tyrosine and threonine is more subjected to post-translational phosphorylation modifications (Figure 6). The number of predicted phosphorylation areas in TtAQPs from group 2 showed higher potential for phosphorylation. Phosphorylation is one of the most important post-translational modifications that affect protein function, durability, and interaction [40,41]. It seems that TtAQP proteins in the second group (PIP2 subfamily) are more involved in cell signaling pathways.

### 3.6. Expression Profile of TtAQPs in Response to Salinity

**Figure 7:**
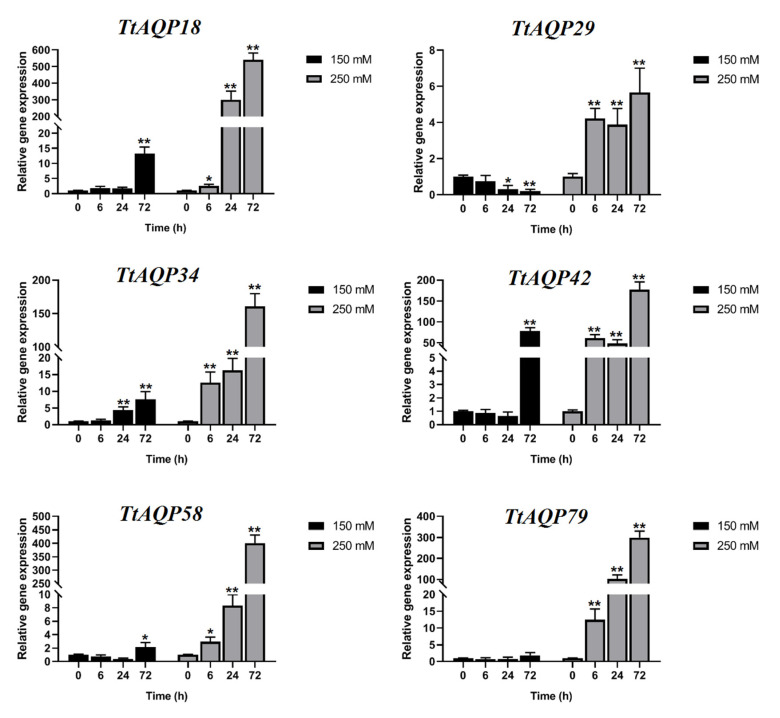
Expression profiles of selected *TtAQP* genes under different concentrations of salinity (150 and 250 mM of NaCl) and four time-points, including 0 (as a normal condition), 6, 24, and 72 h after salt stress. * and ** indicate significant differences between the experimental treatments and control treatment (according to Student’s *t*-test) at *p* < 0.05 and *p* < 0.01, respectively.

The expression pattern of selected genes showed that *TtAQP* genes have different degrees of expression in response to salt stress (Figure 7). Most of the *TtAQP* genes were induced by salinity. In response to 150 mM NaCl treatment, *TtAQP58*, as a *PIP1* gene from group 1, *TtAQP18* and *TtAQP42*, as the *TIP* genes from group 3, and *TtAQP34*, as a *SIP* gene from group 4, were sharply upregulated after 72 h, while two *PIP2* genes from group 2, including *TtAQP29* and *TtAQP79*, were not induced. However, *TtAQP29* showed downregulation after 72 h of 150 mM NaCl. Interestingly, all selected *TtAQPs* were significantly upregulated in response to a high salinity concentration (250 mM of NaCl), and the highest expression levels were observed after 72h of salt stress. Overall, the expression patterns of the six selected *TtAQP* genes in durum wheat showed that *TtAQPs* are mostly involved in the group with late responses to salinity stress.

### 3.7. Promotor Analysis of TtAQP Genes

**Figure 8:**
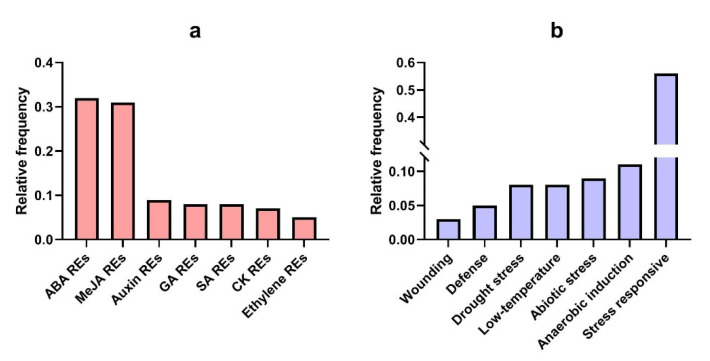
Grouping the cis-regulatory elements related to responsive elements (REs) to phytohormones (**a**), and REs to stress (**b**). Full details are provided in Table S4.

The promoter region of *TtAQP* genes was screened to identify the putative *cis*-regulatory elements. The results disclose that important key regions involved in response to biotic and abiotic stresses, as well as phytohormones, are located in the upstream region of *TtAQP* genes (Figure 8). Moreover, it was found that MYB elements, which are involved in the response to stresses, had the highest frequency in the promoter of *TtAQP* genes (Table S4). Then, CAT-box elements—which are dependent on meristem expression, and ABRE elements, which are regulatory elements involved in response to abscisic acid (ABA)—had the highest frequency in the promoter site of *TtAQP* genes. In addition, MRE elements, which are present in the MYB binding site and play a role in response to light, had the lowest frequency in the promoter site of *TtAQP* genes. Following that, the WUN-motif, which is an element involved in the response to abiotic stresses, and AuxRR-core, which is involved in the response to auxin, had the lowest frequency in the promoter site of *TtAQP* genes. In general, the identified regulatory elements can be classified into four groups, including hormone-responsive, stress, light, and growth elements. The highest of regulatory elements upstream of *TtAQPs* was predicted in the field of stress and hormone-related functions. The presence of these important regulatory elements in the promoter sites indicates that *TtAQP* genes are involved in the response of plants to stress conditions. Overall, *TtAQP* genes showed high potential to participate in various cellular processes, so it is recommended that focus be placed on this gene family in molecular works related to durum wheat breeding.

### 3.8. Protein–Protein Interaction of TtAQPs

**Figure 9:**
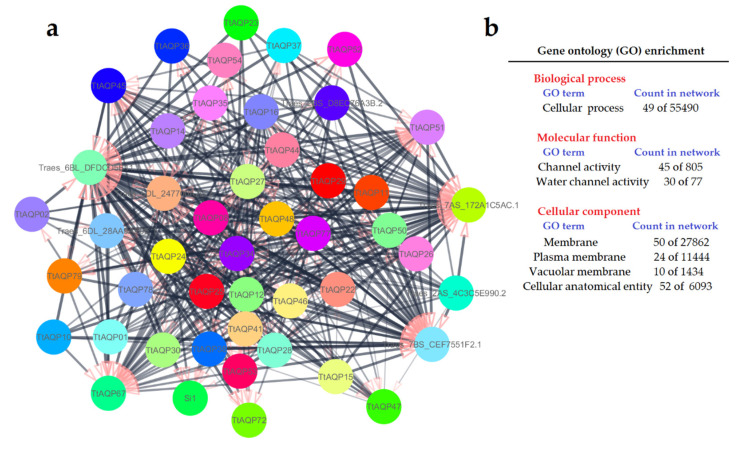
Interaction network of TtAQP proteins (**a**), and list of GO enrichment of proteins presented in the network (**b**). The network was constructed based on their homologs of TtAQPs in *T. aestivum* using the String database.

The interaction network of TtAQPs was constructed based on their orthologues in *T. aestivum* using the STRING database (Figure 9a). According to the predicted network, two PIP1 proteins (TtAQP51 and TtAQP45), two SIP proteins (TtAQP27 and TtAQP34), and two NIP proteins (TtAQP67 and TtAQP47) showed high interaction with other elements in the network (Figure 9a). Moreover, all proteins present in the network were analyzed to identify the significant (FDR < 0.001) gene ontology (GO) terms (Figure 9b). The cellular process in biological process terms, and channel activity and water channel activity in molecular function terms, were significantly enriched. The cellular component GO terms, including the membrane, plasma membrane, vacuolar membrane, and cellular anatomical entity, were significantly enriched. These results reveal that TtAQPs are located in cell membranes and vacuoles and are more involved in the process of transferring water and ions. These results suggest that AQPs interact more with each other, which probably affects their activity levels.

## 4. Discussion

The role of AQPs in regulating cell homeostasis by controlling the flow of water and some ions in the cell membrane is well known [1]. The function of AQP family members has been studied in several plants, although the structure, evolutionary process, and function of this gene family have not been investigated in *T. turgidum*, so far. In the present study, 80 putative *TtAQP* genes were identified and characterized in the genome of *T. turgidum*, as in the first report. Based on previous studies, the number of AQP family members has been variable. For example, 32 *AQPs* were identified in Physic nut [42], 35 in *Arabidopsis* [43], 41 in potato [44], 26 in bamboo [45], 45 in cassava [46], 71 in cotton [47], 67 in *Brassica oleracea* [48], and 51 in flax [49]. The number of *AQPs* was higher in *T. turgidum*; there is a hypothesis that this gene family is more affected by polyploidy and duplication events under the evolution process [50,51]. Additionally, a large number of segmental duplications was observed between TtAQP family members, indicating that segmental duplication has been the main factor responsible for expansion in the TtAQP family under evolution. However, we know that *T. turgidum* underwent an allopolyploid event, which led to extension of the homolog genes in the wheat genome. Moreover, according to the Ka/Ks index, the duplicated members of *TtAQP*s have been under negative selection; as a result, the functional diversity among *TtAQPs* has decreased. The exon/intron number was varied between TtAQP subfamilies, suggesting that each subfamily has had a different evolutionary process and each AQP subfamily was created before the derivation of monocotyledonous and dicotyledonous plants [52]. Furthermore, it has been reported that the intron/exon number can affect gene expression in plant species [51]. According to our phylogenetic analysis, AQP proteins were separated into four main groups, and the subfamilies PIP1 and PIP2 showed more genetic distance than the subfamilies TIP, SIP, and NIP. Previously, it was reported that the NIP subfamily originated from the bacterial genome [53,54]. It seems that this gene family has been subjected to evolutionary pressure and extended via duplication, polyploidy, and horizontal transmission.

Post-translational modifications tightly regulate the activity of aquaporin [55]. In the current study, the PIP2 subfamily proteins showed a high potential for phosphorylation events. PIP2 proteins have higher water channel efficiency than PIP1 proteins [56]. It is hypothesized that phosphorylation affects the channel activity of this subfamily. Phosphorylation is one of the most important post-translational modifications and affects protein function, durability, and interaction [50,57]. Additionally, previous studies revealed that the channel opening of AQPs is influenced by phosphorylation at its C terminal sites [14,58,59]. In addition, it was stated that ethylene can regulate the C terminal phosphorylation of Arabidopsis PIP2;1 (AtPIP2;1) [60]. Moreover, abiotic stresses such as salinity affect the phosphorylation of AtPIP2;1 [61]. Identifying more potential phosphorylation sites in TtAQP proteins could suggest that this subfamily has more channel activity and is more affected by upstream signaling pathways related to phosphorylation events.

The candidate *TtAQP* genes showed diverse expression patterns in response to salinity. In 150 mM NaCl conditions, candidate genes from the PIP2 subfamily showed downregulation, unlike the other selected genes from the PIP1, TIP, and SIP subfamilies. However, with increasing salinity concentration and duration of treatment, all *TtAQPs* were induced, and they showed the highest expression levels. Our results disclose that *TtAQPs* are a part of durum wheat responses to salt stress. The TIP and PIP subfamilies, which are more studied than others [15], are mostly downregulated in response to abiotic stresses such as drought and salt stress [1,16,17]. Additionally, two *PIP* genes in durum wheat, *TdPIP1;1* and *TdPIP2;1*, were identified as being involved in the response to abiotic stresses [62]. In addition, the overexpression of Td*PIP1;1* and Td*PIP2;1* could improve drought and salt tolerance in durum wheat [63]. We speculated that *TtAQPs* are involved in the group of durum wheat with late responses to salinity stress.

## 5. Conclusions

In the present study, 80 putative *TtAQP* genes from the genome of *T. turgidum* were characterized. The results disclosed that subfamilies of TtAQP, including PIP1, PIP2, TIP, NIP, and SIP, have high genetic distances relative to each other. Furthermore, it was found that segmental duplications have played a major role in the extension of the TtAQP family. In addition, we predicted that PIP2 subfamily members have more potential to be influenced by phosphorylation modification and they are probably involved in signaling pathways related to kinases. According to their expression profiles, we conclude that TtAQPs are associated with late responses to salt stress. Overall, we conclude that TtAQPs are diverse proteins, based on their structure, regulatory systems, and expression. The results of this research can be used in further studies related to salt tolerance in durum wheat.

## Figures and Tables

**Table 1:** Summary of AQP properties in *T. turgidum*, *A. thaliana*, *H. vulgare*, *O. sativa*, and *Z. mays*. Full details of physicochemical properties of TtAQPs are shown in Table S2.

Plant Species	Peptide (aa)	Exon	pI	MW (kDa)	GRAVY	Stability
*T. turgidum*	157–392	1–6	4.65–11.05	16.7–41.5	−0.46, 0.94	96%
*A. thaliana*	220–328	1–5	4.50–10.60	23.9–35.4	0.28, 1.02	100%
*H. vulgare*	150–623	1–19	5.48–12.80	16.3–69.2	−0.88, 0.90	71%
*O. sativa*	165–314	1–5	5.91–12.35	17.5–33.5	0.14, 0.92	88%
*Z. mays*	151–564	1–7	5.42–12.11	18.8–62.8	−0.25, 0.95	86%

## Data Availability

Not applicable.

## Supplementary Materials

The following supporting information can be downloaded at: https://www.mdpi.com/article/10.3390/genes14010202/s1, Table S1: List of *TtAQP* gene primers used in real-time PCR; Table S2: Accession number and physicochemical properties of TtAQP gene family members in *T. turgidum*; Table S3: List of the duplicated gene pairs in the TtAQP gene family; Table S4: Functional category of the putative cis-regulatory elements in *TtAQP* promoter.
